# An Epidemiologic Investigation for an Outbreak of Norovirus Infection in a Daycare Center in Gyeonggi-Do, Republic of Korea, 2023

**DOI:** 10.3390/children12020196

**Published:** 2025-02-06

**Authors:** Sang-Jin Lee, Jieun Jang, Kwan Lee

**Affiliations:** 1 Pyeongtaek-si Public Health Center, Pyeongtaek 17901, Republic of Korea; kiki5869@korea.kr; 2 Department of Preventive Medicine, College of Medicine, Dongguk University, Gyeongju 13557, Republic of Korea; jieunjang@dongguk.ac.kr

**Keywords:** diarrhea, children, epidemiologic investigation, norovirus infection, daycare center

## Abstract

**Background/Objectives**: Norovirus is one of the primary causes of foodborne illness in South Korea. This study aimed to identify the cause of a norovirus outbreak in a daycare center in a city in February 2023 and to prevent further spread through an epidemiologic investigation. **Methods**: A total of 93 individuals, including daycare staff, children, and kitchen staff, were included. A field epidemiologic investigation was conducted, consisting of case definition, collection of environmental and preserved food samples, and human specimens, along with interviews. A matched case-control study (1:3 ratio) was designed to analyze risk factors, and a statistical analysis, including odds ratios with 95% confidence intervals and chi-square tests, was performed to identify associations between food consumption and infection. Person-to-person transmission was also assessed through epidemiological data analysis. **Results**: Among the 93 individuals, 16 (17.2%) were identified as cases, of which nine (9.7%) were confirmed cases. The same genotype of norovirus (GII) was detected in eight human samples. No significant association was found between specific food items and infection. The epidemic curve and transmission network analysis suggested that the primary mode of transmission was person-to-person contact within the daycare center. **Conclusions**: This study highlights the critical role of hygiene practices in daycare settings to prevent person-to-person transmission of norovirus. Regular public health education, environmental disinfection, and early symptom recognition are essential preventive measures.

## 1. Introduction

The World Health Organization (WHO) and the Korea Disease Control and Prevention Agency (KDCA) define foodborne illnesses as gastrointestinal infections caused by bacteria, viruses, parasites, or chemicals entering the body through contaminated food, with symptoms such as diarrhea, abdominal pain, and vomiting [1,2]. Foodborne infections are broadly classified into two types: emetic and diarrheal. The emetic type primarily causes vomiting and includes illnesses caused by norovirus and rotavirus, while the diarrheal type mainly causes diarrhea and includes infections caused by Salmonella and *Escherichia coli (E. coli).* Norovirus is one of the most common causes of foodborne illnesses in South Korea, with the majority of cases occurring during the winter months and frequent outbreaks reported in mass-catering facilities [3]. The virus primarily spreads through the fecal-oral route, but can also be transmitted via vomiting, consumption of contaminated water, aerosolized fecal matter or vomit from infected individuals, or direct contact with infected persons. These diverse transmission pathways contribute to the high likelihood of group outbreaks [4]. Notably, as few as 100 viral particles can cause an infection [5]. Studies indicate that while norovirus can affect individuals of all ages, symptomatic rates are higher among young children in settings such as kindergartens and elderly individuals over 65 years of age [6]. Additionally, symptoms may become severe or chronic in some cases [7]. Norovirus is a common cause of pediatric gastrointestinal illnesses, which, although generally self-limiting, pose significant risks to children with underlying chronic conditions [8]. Consequently, it is essential to promptly identify sources of infection and prevent spread within groups, particularly during outbreaks involving children or elderly individuals. Recently, an outbreak of norovirus infections has been prevalent in daycare centers in South Korea. Particularly in daycare settings, where children live in close quarters and have difficulty maintaining personal hygiene, infections can spread rapidly. Moreover, cases in children tend to be more severe compared to adults. Therefore, documenting and researching outbreaks in daycare centers are crucial for both prevention and further study.

Norovirus is a major cause of foodborne illness in South Korea, accounting for 94.9% of viral foodborne illnesses reported between 2010 and 2019. In 2023, 5926 cases were reported, and 16,784 individuals were treated for norovirus-related gastroenteritis in the same year [9,10,11]. Additionally, according to the U.S. Centers for Disease Control and Prevention (CDC), over 2.7 million people seek medical care annually due to norovirus infections [12]. These figures underscore the significant public health burden posed by norovirus, particularly in communal environments such as daycare centers, highlighting the need for effective preventive measures. Despite its high burden, gaps remain in understanding the specific transmission dynamics of norovirus within communal settings like daycare centers. Previous studies have primarily focused on foodborne transmission, with limited research available on person-to-person transmission in daycare environments. Effective outbreak management requires identifying the primary transmission routes and implementing targeted interventions to prevent further spread. This study aims to fill this gap by investigating a norovirus outbreak in a daycare center in South Korea, focusing on person-to-person transmission patterns, assessing key risk factors, and proposing preventive strategies.

## 2. Materials and Methods

### 2.1. Outbreak Notification

On 9th February (Thursday), at approximately 9:30 AM, the director of a daycare center reported to a city health authority that multiple cases of vomiting and diarrhea had occurred among the children. Following confirmation that one child (Child D) tested positive for norovirus on laboratory tests following admission to a medical facility, the health authorities suspected a norovirus-related outbreak at the daycare center. Consequently, an epidemiological investigation was initiated that afternoon.

### 2.2. Outbreak Setting

The daycare center comprised 93 members, including 68 children, 13 teaching staff, 3 kitchen staff, and 9 additional personnel. The children were organized into eight classes within a two-story building. The first floor contained the staff cafeteria, kitchen, health room, administrative office, integrated care room, and Classes I and II, which accommodated the youngest children. The second floor housed another administrative office, the staff lounge, and the remaining six classrooms. Nine children under the age of three stayed on the first floor, while 59 children aged three to seven occupied classrooms on the second floor. Teaching staff had offices on both floors but spent most of their time caring for children in their designated classrooms. Administrative staff primarily worked in offices, and kitchen staff remained mostly in the kitchen and staff cafeteria areas.

Meals were served three times a day at the daycare center, with all individuals consuming the same menu. Additional staff dined in the first-floor staff cafeteria while children ate in their respective classrooms, accompanied by teaching staff. During mealtime, teaching staff collected food carts from the kitchen and distributed meals to the children in their classrooms.

### 2.3. Case Definitions

Cases were defined as children attending the daycare or staff members working there between 3 February and 7 February 2023, who consumed meals during this period and experienced at least two episodes of diarrhea (looser than normal stools) or vomiting within 24 h, or symptoms persisting for more than two days, or tested positive for the pathogen in laboratory tests.

### 2.4. Study Design

A case-control design was used to identify the source of the outbreak. Controls were selected from children and staff members who were present at the daycare center during the same period and consumed the same meals as the cases but did not develop symptoms. For children, symptomatic and asymptomatic individuals were selected from the same class, while for adults, symptomatic individuals were matched with other asymptomatic individuals. A 1:3 ratio of cases to controls was used.

### 2.5. Data Collection

Data were collected using the epidemiologic investigation form specified in the “2022 Waterborne and Food-borne Infectious Disease Management Guidelines” through individual interviews [2]. The form included information on gender, birthdate, symptoms, symptom frequency and duration, and food consumption history. The daycare center director provided a list of children and staff, along with facility and environmental information. Additional information regarding the daycare and home environments was obtained through interviews with staff and parents. Details related to food consumption have been presented in the results section.

### 2.6. Laboratory Testing

On 9 February 2023, public health personnel collected human and environmental specimens from symptomatic patients, staff members who had close contact with symptomatic children, and food service workers, in accordance with the 2022 Waterborne and Foodborne Infectious Disease Management Guidelines, and submitted them to the Gyeonggi-do Institute of Health and Environment for analysis. A total of 17 human specimens were collected using rectal swabs, and five types of viruses, including norovirus, were analyzed using RT-PCR, while ten types of bacteria, including *Escherichia coli (E. coli)*, were identified through culture tests. Additionally, 33 preserved food samples and 14 environmental samples were tested for ten types of bacteria and norovirus. However, as the parents of asymptomatic children did not consent to testing, specimens were collected only from symptomatic children.

### 2.7. Investigation Procedure

Upon receiving the report from the daycare center director at 9:30 AM on 9 February (Thursday), health authorities dispatched an epidemiologic investigator, an infectious disease officer, and a food safety team member to the site at approximately 2:00 PM the same day. The team conducted an epidemiologic investigation and collected specimens. The on-site investigation was carried out collaboratively by the health authorities and the city’s food safety team. Human specimens were collected from both symptomatic individuals and kitchen staff, totaling 17 samples (13 from symptomatic individuals and 4 from asymptomatic individuals). The facility manager and director conducted additional investigations to identify common sources of exposure beyond meal-related environments within the daycare center. Individual dietary exposure was assessed through personal epidemiological surveys. Telephone interviews were conducted with staff and parents, and home visits were carried out when further investigation was deemed necessary.

Environmental samples were collected from the kitchen and areas associated with symptomatic children and staff. Preserved food samples from 3 February (Friday) to 7 February (Tuesday) were also gathered for testing. Following the on-site investigation, the daycare center director was instructed to disinfect all areas of the facility, and symptomatic individuals were advised to return only after three days of symptom resolution. Staff and children were also directed to maintain regular hand hygiene and continue environmental disinfection efforts.

### 2.8. Statistical Analysis

Statistical analyses were conducted using Microsoft Excel 2016 and Epi Info (V7.2.6.0., CDC in Atlanta, GA, USA) to calculate the odds ratio (OR) and 95% confidence intervals (CIs) for each meal item, with a significance threshold of *p* < 0.05. A 1:3 ratio of cases to controls was used, pairing adults with adults and children with children, resulting in 16 cases and 48 controls. The analysis was performed based on food consumption.

### 2.9. Ethics

This study adhered to the regulations set forth by the KDCA, Ministry of Health and Welfare (MoHW), and Gyeonggi-do Provincial Government, in accordance with the Bioethics and Safety Act (Article 15) and the Infectious Disease Control and Prevention Act (Article 18). Consequently, additional ethical approval from an Institutional Review Board (IRB) or Ethics Committee was not required. All personal information was removed prior to data analysis. An epidemiological investigation was conducted with the individual consent of each parent or guardian, and personal information can be collected in the event of an outbreak of a communicable disease in accordance with the Infectious Disease Control and Prevention Act of Korea. All personal information was removed prior to analysis. Additionally, the reason for the exemption from IRB approval is based on legal grounds for prompt public health response.

## 3. Results

The index case occurred on 5 February 2023 (Sunday) at approximately 1:00 PM, when a child developed symptoms at home. The final case was reported on 12 February 2023, bringing the total to 16 reported cases. Norovirus was detected in specimens from nine individuals, while all three kitchen staff members tested negative. Additionally, all environmental samples were negative for norovirus.

### 3.1. Epidemic Curve

**Figure 1:**
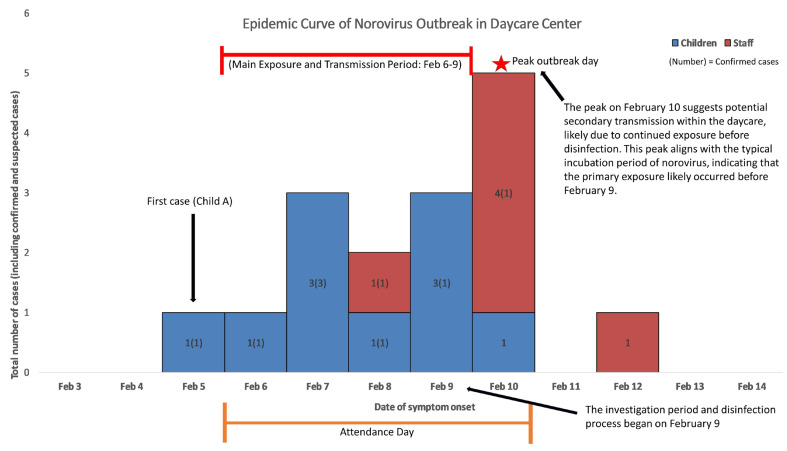
Distribution of clinical cases by day of onset in children and staff (Epidemic curve).

Following the first symptomatic case on 5 February, additional cases were reported daily until 12 February, except for 11 February. The epidemic curve shows a gradual increase in cases, peaking on February 10 with five new cases reported. This pattern suggests the possibility of ongoing secondary transmission within the daycare center rather than a single exposure event, with additional cases likely arising due to continued exposure through environmental contamination or close human contact over time. Notably, the peak on 10 February likely reflects the incubation period of norovirus, indicating that primary exposure most likely occurred before 9 February. Figure 1 visually presents the temporal distribution and clustering pattern of infection cases.

### 3.2. Results of Laboratory Tests of the Specimens

Of the 17 human specimens submitted for testing, 8 tested positive for norovirus, all of which were identified as the same GII genotype (Norovirus GII). Among the positive cases, six were children and two were staff members, and additional positive cases were confirmed at an external hospital. Furthermore, no norovirus was detected in any of the 47 environmental samples, including 33 preserved food samples, 4 kitchen samples, and 10 environmental samples (Table 1). The identification of Norovirus GII confirms the causative pathogen of this outbreak.

### 3.3. Laboratory Findings

No pathogens were detected in the preserved food and food service workers’ specimens. Considering that no additional cases occurred after five patients were reported on the 10th and one additional staff case on the 12th, it is highly likely that the patients who appeared after the 10th were infected through spatial or person-to-person transmission before the disinfection conducted on the 9th. After an incubation period, symptoms began to appear from the 10th. Additionally, the confirmation of Norovirus GII in human specimens indicates the possibility of transmission through person-to-person contact or environmental exposure. Given that cases continued to emerge within the same space, it is reasonable to assume that if disinfection had not been conducted, viruses would likely have been detected in the environmental samples.

### 3.4. Incidence Rate

Among the 93 individuals, 16 (17.2%) were identified as cases, of which nine (9.7%) were confirmed cases (Table 2).

### 3.5. Risk Factor Analysis

To identify the causative food item, food consumption data from 3 February (Friday) to 7 February (Tuesday) were analyzed. The 16 cases noted on the epidemiologic curve (Epi curve) were compared to 48 controls without symptoms to assess associations with food consumption. Statistical analysis was conducted using a significance level of *p* < 0.05 and a 95% confidence interval. However, no statistically significant associations between food consumption and cases were identified. Among the 33 preserved food items analyzed, none showed a statistically significant association with the outbreak. Therefore, for clarity and conciseness, only the three items with the lowest *p*-values are presented in Table 3, while the remaining items have been omitted. In addition, no common food consumption was found among the cases outside the meals provided at the daycare center. In South Korea, it is common for milk and kimchi to be included in daily institutional meal plans in daycare centers, schools, and similar facilities.

According to studies, norovirus is a highly transmissible infectious disease that spreads primarily through person-to-person contact. Therefore, in this outbreak, human-to-human transmission is likely to have been the primary mode of infection rather than contaminated food.

**Table 3:** Statistical analysis of food consumption (top 3 items with the lowest *p*-values among 33 preserved food items) (*n* = 64).

Date	Food Items	Cases		Controls		*p*	OR (95% CI)
		Exposed	Unexposed	Exposed	Unexposed		
3 February	Milk	16	0	41	7	0.41	5.46 (0.29–101.98)
	Stir-fried fernbrake	12	4	44	4	0.19	0.27 (0.06–1.25)
	Kimchi (Napa cabbage)	15	1	45	3	0.55	1.00 (0.10–10.35)
6 February	Abalone and vegetable porridge	14	2	36	12	0.49	2.33 (0.46–11.78)
	Fish cake soup	14	2	43	5	0.82	0.81 (0.14–4.67)
	Seasoned bean sprouts	15	1	45	3	0.55	1.00 (0.10–10.35)
7 February	Fermented soybean paste stew	15	1	44	4	0.79	1.36 (0.14–13.18)
	Sweet pumpkin porridge	14	2	43	5	0.82	0.81 (0.14–4.67)
	White kimchi	14	2	46	2	0.55	0.30 (0.04–2.36)

Other items not shown did not present significant associations (*p* > 0.05).

### 3.6. Estimating Transmission Mode

**Figure 2:**
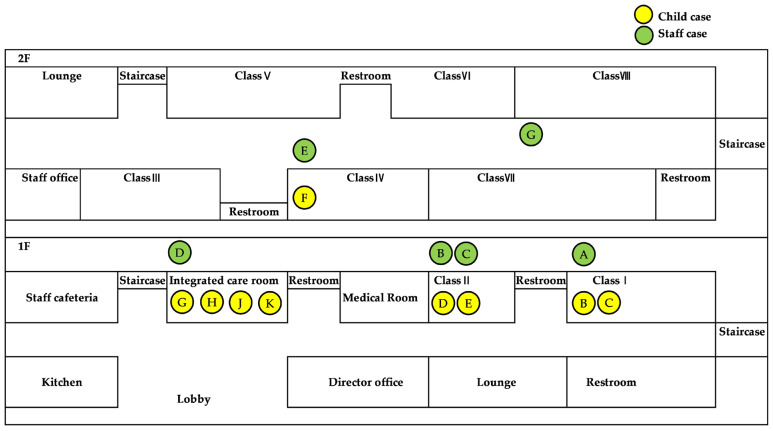
Presumed risk exposure locations and facility overview within the daycare center.

**Figure 3:**
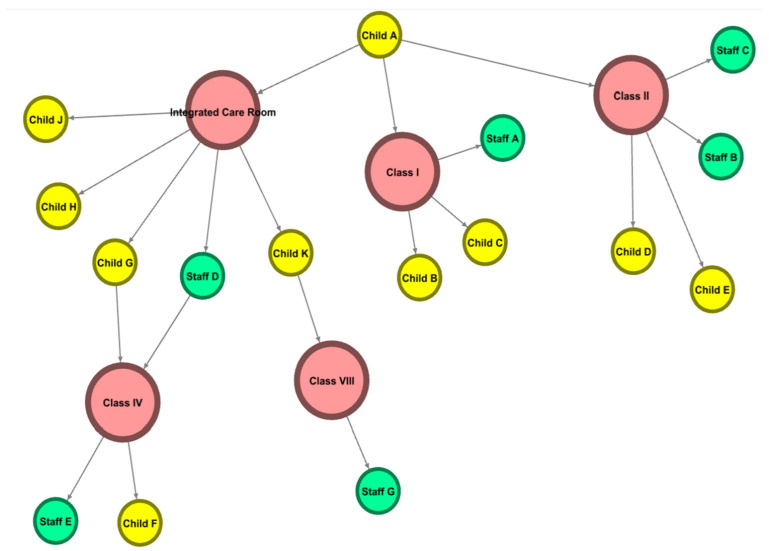
Case summary and presumed risk exposure locations in the daycare center.

Based on individual epidemiological interviews, the estimated exposure sites and transmission networks within the daycare facility are shown in Figure 2 and Figure 3, and Table 4. According to studies, close contact in daycare environments has been identified as a major route of norovirus transmission between individuals [10,11]. Symptom onset dates and presumed risk factors were also considered to establish causality. Child A, who belonged to Class I, developed symptoms on 5 February and attended daycare on 6 February, initiating the transmission. After attending daycare, Child A spent time in the integrated care room and remained in Class I throughout the day, having close contact with staff and other children. Inadequate hand hygiene by staff and the management of vomit and feces within the facility likely played a significant role in virus transmission.

The initial cases began in the integrated care room, Class I, and Class II. Previous studies have reported that shared use of restrooms and communal spaces can increase the risk of norovirus transmission in daycare environments [10,11]. According to the transmission network analysis, secondary transmission to Classes IV and VIII likely occurred through contact with Staff Member D, who worked in the integrated care room. Given that Staff Member D was responsible for diaper changes and close interactions with children, direct contact transmission is considered highly likely.

Furthermore, Staff Member D, who handled Child A’s feces in the integrated care room, likely transmitted the virus to Child F from Class III on the second floor due to inadequate hand hygiene. The possibility of transmission through aerosols and fomites, such as vomit and contaminated surfaces, has also been documented in previous studies, supporting the likelihood of secondary infections occurring in shared spaces [10,11]. Child K, who used the integrated care room, may have subsequently transmitted the virus to Staff Member G during classroom activities.

**Table 4:** Summary of symptom onset dates and estimated risk factors for children and staff (*n* = 16).

No.	Child/ Staff	Cases	Onset	Test Result	Risk Factor
1	Child	Child A	5 February 13:00	Norovirus GII +	Unknown/Primary case
2		Child B	7 February 23:30	Norovirus GII +	Class I
3		Child C	7 February 20:00	Norovirus GII +	Class I
4		Child D	6 February 15:00	Norovirus * +	Class II
5		Child E	9 February 5:00	Norovirus GII +	Class II
6		Child F	8 February 5:00	Norovirus GII +	Class IV
7		Child G	9 February 8:00		Integrated Care Room
8		Child H	9 February 5:00		Integrated Care Room
9		Child J	7 February 16:00	Norovirus GII +	Integrated Care Room
10		Child K	10 February 20:00		Integrated Care Room
11	Staff	Staff A	8 February 15:00	Norovirus GII +	Class I
12		Staff B	10 February 8:00		Class II
13		Staff C	10 February 10:00		Class II
14		Staff D	10 February 5:00	Norovirus GII +	Integrated Care Room
15		Staff E	10 February 8:00		Class IV
16		Staff G	12 February 8:00		Class VIII

* The norovirus-positive result for Child D was confirmed at an external hospital. Since the sample was not submitted for genetic analysis, the genogroup could not be determined.

## 4. Discussion

If person-to-person transmission via contact was the primary cause of this outbreak, norovirus would likely have been detected in environmental samples from locations such as classrooms and bathrooms. It is presumed that no pathogens were detected in the environmental samples due to the complete disinfection of all areas within the daycare center prior to the epidemiological investigation. However, it is also possible that the number and volume of samples, or the selection of appropriate sampling targets, may have been insufficient. This study confirms that norovirus outbreaks in daycare centers can spread rapidly through person-to-person contact, emphasizing the importance of hygiene practices and prompt disinfection measures to prevent secondary infections. In general, the norovirus infection often leads to outbreaks through the consumption of contaminated water or food. However, when personal hygiene measures, such as handwashing, and environmental hygiene practices, such as disinfection, are inadequate, person-to-person transmission can easily occur. The findings highlight that comprehensive disinfection efforts for potentially infectious vomitus or fecal matter were effective in halting further transmission within the daycare. Implementing strict hygiene protocols, such as handwashing and environmental cleaning, is crucial to prevent future outbreaks in such settings. Most cases occurred before 10 February. Considering the incubation period (12–48 h), it is likely that transmission occurred through co-residence in the same space or close contact before disinfection, following the attendance of the initial case. In this epidemiological investigation, “contact transmission” encompasses not only physical contact but also infection through co-presence within the same space. This interpretation aligns with findings from other studies where contact transmission was identified as a major pathway of infection under similar circumstances. If environmental samples had been collected before disinfection, it would have been possible to identify the pathogens, which would have greatly contributed to establishing causality. No statistically significant food items were identified in the food consumption history analysis. No associations were found in the food consumption records or kitchen environment samples, and norovirus was not detected in food service personnel who had no direct contact with the children and staff. Considering that the cases clustered within the same spaces and the identical genotype of the detected norovirus, it is reasonable to conclude that the outbreak was more likely caused by person-to-person transmission rather than foodborne infection. Due to the inability to investigate some children, a case-control design was chosen. However, given the small sample size in the statistical analysis of 16 cases and 48 controls, it cannot be ruled out that the lack of significance may be attributable to the limited sample size. This study has several limitations. First, the small sample size (16 cases and 48 controls) may have limited the statistical power to detect significant associations with food consumption. Second, recall bias could have influenced the accuracy of exposure history collected from parents and staff, particularly for commonly consumed items such as rice and kimchi. Third, due to the homogeneous nature of daycare meals, identifying specific sources of contamination was challenging. Fourth, norovirus was not detected in environmental samples. However, a comprehensive assessment of the living spaces and objects used by children was not feasible. Moreover, environmental disinfection was conducted prior to the epidemiological investigation, which likely hindered the detection of norovirus in the environment. Future studies should incorporate larger sample sizes, detailed dietary exposure assessments, and more thorough environmental sample collection process to strengthen the reliability of findings. The limited statistical power resulting from the small sample size emphasizes the need for careful interpretation of the results. Future studies should consider a larger cohort to better assess potential associations and identify causative factors with greater confidence. The causative pathogen in this outbreak was confirmed to be Norovirus GII. Over the past five years (2019–2023), norovirus was the most prevalent causative virus of gastrointestinal infections in South Korea, with the GII genogroup being the most dominant genotype [9]. The absence of norovirus in preserved food samples, combined with the fact that all individuals within the same age group consumed the same meals while only children and staff were in close contact with specific affected individuals, indicates that the outbreak was unlikely to have been caused by foodborne transmission. 

Considering that Child A exhibited symptoms on the weekend (Sunday) and then attended daycare, with subsequent cases emerging in close proximity, it is plausible that Child A was initially infected from an unknown source outside the daycare and later transmitted the infection through contact within the facility. Studies have demonstrated that norovirus transmission can occur rapidly among individuals sharing the same space and in close proximity. In a study similar to this research, a study conducted in a kindergarten setting found that norovirus transmission between individuals in close contact within the same space occurred rapidly [10,11]. Therefore, in this study, since food was not identified as the source, and cases continued to emerge in adjacent spaces, it was reasonable to infer that person-to-person transmission occurred through contact and shared space. Referring to previous studies where transmission within a space occurred rapidly via aerosols or vomiting, this study also aligns with such findings. Additionally, according to a study by Jing Wang (2023), environmental disinfection was shown to effectively reduce person-to-person transmission of norovirus [10]. Following the complete disinfection on the 9th, no further group outbreaks from contact within the space occurred after the 10th, except for cases that arose from contact prior to disinfection. Transmission through aerosols, such as vomit and fecal aerosols, is also a possible and critical mode of contamination for norovirus [11]. Research on the spread of norovirus in indoor environments indicates that the virus can be transmitted through contact with vomiting residue, dust, or airborne particles [12]. Environmental factors, including the area, volume, and ventilation of indoor spaces, have also been shown to influence the dynamics of norovirus outbreaks [13]. As norovirus spreads easily in contaminated environments, it is essential to disinfect areas prone to human exposure, such as bathrooms and living spaces, to prevent its transmission [14]. Research on norovirus persistence suggests that the virus can remain infectious on inert surfaces, including stainless steel and polyvinyl chloride, for extended periods, thereby retaining its infectivity [15]. Additionally, norovirus can be transmitted asymptomatically, meaning that individuals without symptoms can still spread the virus. To prevent such transmission, daycare staff should promptly notify health authorities upon recognizing symptomatic children, while parents should communicate any symptoms observed in their child to the daycare staff before attending. This proactive communication would significantly help in preventing outbreaks. Additionally, it is crucial to conduct such educational programs regularly. Limiting activities such as handling food through hand contact or providing personal care to others can significantly reduce person-to-person transmission [10,16,17,18].

Had symptomatic children, such as Child A and others who returned immediately after symptom resolution, been kept at home, widespread transmission within the facility might have been prevented, thereby minimizing both human and administrative losses from the outbreak. This highlights the importance of educating daycare personnel to ensure that children with suspected symptoms of infection remain at home. Parents and staff should also be well-informed through internal educational programs. Furthermore, enhancing hand hygiene practices and implementing robust surface disinfection protocols as environmental interventions can reduce norovirus surface contamination [19]. Moreover, these hygiene measures can result in significant cost savings for communal facilities [20].

Additional steps, such as restricting contact with external personnel following an outbreak, may help prevent subsequent cases [21]. The identification of the causative pathogen and the resolution of an infectious disease outbreak highlight the need for local health departments to prioritize infectious disease prevention education, oversee the management of facilities within their jurisdiction, and develop monitoring strategies for students [22]. Similarly, food safety teams should conduct regular inspections and offer guidance to prevent foodborne illnesses in facilities that provide communal dining services. Future research should focus on conducting larger cohort studies to better understand the epidemiological characteristics of norovirus outbreaks in daycare settings. Future epidemiological investigations of norovirus outbreaks should aim to conduct cohort studies encompassing the entire exposed population whenever possible. Additionally, environmental disinfection should be avoided before the epidemiological investigation, and environmental samples should be collected from various objects and locations. Additionally, more detailed environmental sampling protocols should be developed to capture potential sources of contamination that may have been missed in this study. Expanding surveillance efforts to other communal environments, such as schools and nursing homes, would provide valuable insights into norovirus transmission dynamics.

## 5. Conclusions

In conclusion, the primary cause of the outbreak at the daycare center was attributed to Child A, with the outbreak confirmed as a foodborne norovirus illness transmitted person-to-person. Waterborne and foodborne infectious diseases are common in environments where children, particularly infants, live collectively, such as daycare centers. Therefore, in such densely populated environments, strict management of personal hygiene and environmental sanitation is essential to prevent outbreaks.

In dense environments such as daycare centers, group infections can rapidly occur through contact. To prevent this, not only basic hygiene practices but also preventive activities through public health education are crucial.

Norovirus is highly transmissible among children, highlighting the importance of close cooperation between households and daycare centers. Parents should promptly notify the daycare center if their child exhibits suspected symptoms such as diarrhea or vomiting. Similarly, daycare centers should quickly identify children with such symptoms and assess their attendance eligibility based on internal guidelines. In addition, given the challenges of conducting direct interviews with younger children, parental cooperation and heightened awareness of infectious diseases through education are essential. This approach can streamline epidemiological investigations, allowing for rapid identification of transmission routes and the effective containment of further spread. Education for both daycare centers and parents is crucial to ensure that children with symptoms do not attend daycare. Public health authorities should make infectious disease training mandatory for a small group of staff members and consistently offer educational programs to parents to raise awareness and promote vigilance regarding these infections.

Moreover, in environments where many children cannot manage their toileting, such as daycare centers, thorough diaper-changing practices and strict hygiene management are essential. The daycare center implemented integrated care, which led to a high frequency of contact between children from different groups. The initial cases predominantly involved the youngest children in the daycare, who required close care from staff. These children, who were unable to manage personal hygiene independently, such as during meals and diaper changes, contributed to the rapid spread of infection. While the disinfecting practices and hygiene behaviors of the staff were important, the most crucial aspect of public health prevention education was the prompt reporting of cases to relevant personnel or parents and ensuring the child does not attend daycare. Particularly when children exhibit symptoms, it is critical that they do not attend daycare; however, due to various circumstances, strict management of this measure is often challenging. Children presenting with symptoms such as diarrhea should be immediately reported and managed through strengthened surveillance systems. By implementing these multifaceted efforts, norovirus outbreaks can be effectively prevented, contributing to enhanced infectious disease management in communal settings like daycare centers.

## Figures and Tables

**Table 1:** Laboratory test results of environmental specimens.

Specimen	Specimen Type	Number of Samples	Test Result
Reserved Food	White rice and 32 other types	33	Negative
Kitchen Utensils	Refrigerator, valves, warehouse handles, etc.	4	Negative
Environmental Specimens	Environmental specimens within classrooms where cases occurred (toys, tables, restrooms, etc.)	10	Negative

**Table 2:** Attack rates and number of patients among teachers, children, and other staff at the daycare center.

Category	Total (*n* = 93)	Staff (*n* = 13)	Child (*n* = 68)	Other Staff (*n* = 12)
Cases (*n*, %)	16 (17.2%)	6 (46.2%)	10 (14.7%)	0 (0.0%)
Confirmed cases (*n*, %)	9 (9.7%)	2 (15.4%)	7 (10.3%)	0 (0.0%)

## Data Availability

Data available on request due to privacy.
